# Enhanced Sensitivity in D-Shaped Optical Fiber SPR Sensor via Ag-α-Fe_2_O_3_ Grating

**DOI:** 10.3390/mi17020183

**Published:** 2026-01-29

**Authors:** Shuai Yuan, Bingyang Yuan, Jiu Deng

**Affiliations:** 1 School of Health and Life Sciences, University of Health and Rehabilitation Sciences, Qingdao 266113, China; yuanby@hrbeu.edu.cn; 2 Ocean Decade International Cooperation Center (ODCC), Qingdao 266520, China; 3 College of Shipbuilding Engineering, Harbin Engineering University, Harbin 150001, China

**Keywords:** optical fiber sensors, surface plasmon resonance, hematite, simulation

## Abstract

The development of high-performance optical fiber sensors based on surface plasmon resonance (SPR) represents a significant advancement in precision detection technology, particularly for biomedical and environmental monitoring applications requiring real-time response and minimal sample consumption. This research conducts a systematic numerical investigation of a D-shaped fiber SPR sensor incorporating an optimized silver-hematite (Ag-α-Fe_2_O_3_) composite grating structure. Through comprehensive finite element simulations and parameter analysis, we demonstrate that controlling the silver layer thickness at 45 nm while maintaining the α-Fe_2_O_3_ thickness at 12 nm achieves optimal electric field confinement. The grating gap width optimization at 30 nm enables maximum sensitivity through enhanced localized surface plasmon resonance effects, while the residual cladding thickness of 0.5 μm provides the ideal balance between detection accuracy and sensitivity. The research establishes fundamental design principles for high-performance SPR sensors by elucidating the critical relationships between geometric parameters and sensing characteristics, providing valuable insights for developing next-generation sensors with enhanced performance for advanced sensing applications in environmental monitoring and medical diagnostics.

## 1. Introduction

Microscale detection technology has emerged as a pivotal advancement in modern analytical science, offering exceptional capabilities for minimal sample consumption, rapid response times, and high-throughput analysis [1,2,3]. These attributes render it particularly valuable for biomedical diagnostics and environmental monitoring applications [4,5,6]. The integration of microfluidic platforms with optical sensing methodologies has further enabled sophisticated real-time in situ detection schemes with enhanced precision. Among them, surface-enhanced Raman scattering, which exploits the localized surface plasmon resonance of noble metal nanostructures to amplify Raman signals by several orders of magnitude, has emerged as a powerful tool for trace-molecule analysis [7,8]. Moreover, surface plasmon resonance (SPR) technology has also gained prominence as a powerful label-free detection technique capable of monitoring molecular interactions through refractive index variations with remarkable sensitivity [9]. This approach has demonstrated significant potential across diverse fields including environmental surveillance [10], biomolecular diagnostics [11], and clinical testing [12] due to its label-free operation and real-time monitoring capabilities.

Conventional SPR sensors typically employ prism-coupled configurations that, despite their high sensitivity, suffer from inherent limitations such as bulky instrumentation and substantial costs, thereby restricting their practical deployment in field applications. In contrast, fiber-optic SPR sensors offer distinct advantages including compact dimensions, mechanical flexibility, and cost-effectiveness while maintaining the fundamental capability to detect refractive index changes with nanoscale precision through resonance condition modulation at the metal-dielectric interface [13]. This unique combination of characteristics positions fiber-optic SPR sensors as an ideal technological platform for substance detection in microscale environments [14].

The operational principle of SPR sensors relies on the excitation of surface plasmon waves at the metal-dielectric boundary, where resonance energy transfer occurs when the propagation constant of incident light matches that of surface plasmons [15]. This phenomenon generates a characteristic dip in the reflection spectrum, with its spectral position exhibiting high sensitivity to minute changes in the surrounding refractive index. Recent advancements in fiber-optic SPR designs have focused on structural innovations and material engineering to optimize sensor performance, including the development of specialized geometries such as D-shaped fibers [16], photonic crystal fibers [17], and grating-assisted configurations [18]. Among these, grating-assisted configurations are particularly noteworthy. By introducing periodic metallic nanostructures, they can efficiently excite localized surface plasmon resonances, leading to significantly enhanced electromagnetic field confinement at the metal-dielectric interface. This strong field enhancement is a key factor in achieving superior sensitivity. However, the performance of such grating-based SPR sensors is critically dependent on their precise geometric parameters, especially the dimensions of the metallic grating elements themselves. These developments have substantially improved detection capabilities while addressing practical challenges associated with traditional SPR implementations.

The evolution of optical fiber-based SPR refractive index sensors has become a research focus due to their structural adaptability and electromagnetic interference immunity. Through sophisticated optical fiber architectures and composite material systems, researchers have achieved remarkable progress in enhancing sensor performance. In D-shaped optical fiber configurations, Osamah et al. [19] demonstrated a symmetrical grating structure that achieved 2 μm/RIU (Refractive Index Unit) sensitivity at RI (Refractive Index) = 1.5 through optimization of gold layer thickness and grating depth, albeit within a limited detection range (RI = 1–1.5). More notably, Mu et al. [20] developed an Au/Ti_3_C_2_T_x_-MXene hybrid layer D-shaped photonic crystal fiber sensor that achieved exceptional performance metrics, reaching an average sensitivity of 48.12 μm/RIU and a maximum sensitivity of 64.6 μm/RIU within the refractive index range of 1.390–1.415. This enhancement is attributed to MXene materials’ extensive specific surface area and enhanced plasmonic coupling effects. Material innovations have further advanced SPR sensor capabilities. Kadhim et al. [21] implemented an Ag-α-Fe_2_O_3_ grating structure, in which Fe_2_O_3_ has high chemical stability and durability [22]. The results show that a 12 nm hematite coating effectively mitigated silver oxidation while achieving 6.4 μm/RIU sensitivity within RI = 1.33–1.39. Simultaneously, Dogan et al. [23] designed a MoS_2_/graphene multilayer sensor that attained 11.78 μm/RIU sensitivity (RI = 1.33–1.40), representing a 98.7% improvement over conventional silver-coated sensors and underscoring the considerable potential of two-dimensional materials in enhancing SPR effects. Additionally, Yan et al. [24] proposed a V-shaped photonic crystal fiber sensor that achieved 14.78 μm/RIU average sensitivity in higher refractive index regimes (1.47–1.52), providing new perspectives for high-refractive-index liquid detection.

Despite these remarkable advances, the field faces persistent challenges including insufficient systematic parameter optimization of critical factors like silver grating thickness and air gap width matching. To address this gap with a promising material platform, we focus on an Ag–α-Fe_2_O_3_ composite grating. Here, Ag is chosen for its lower optical loss and sharper resonance than Au, favoring high sensitivity, while α-Fe_2_O_3_ provides a higher refractive index for field modulation and serves as a protective layer against oxidation. However, even with such composite materials, a deep and systematic investigation into the interplay and optimization of fundamental geometric parameters remains essential. These parameters directly govern the balance between LSPR strength, mode coupling efficiency, and optical losses, ultimately determining the sensor’s sensitivity, detection accuracy, and overall figure of merit. Accordingly, this research contributes to this evolving field by presenting a numerical investigation of a D-shaped fiber SPR sensor with an Ag–α-Fe_2_O_3_ grating structure. Through systematic finite element analysis, we explore the influence of critical parameters, including structural dimensions such as silver layer thickness, grating gap width, hematite thickness, and residual cladding thickness, on sensor performance metrics. The simulation framework incorporates comprehensive modeling of electromagnetic field distribution, loss spectrum characteristics, and phase-matching conditions to establish a robust theoretical foundation for subsequent practical implementation. This study aims to elucidate, through systematic finite-element analysis, the influence mechanisms of key geometric parameters within the Ag-α-Fe_2_O_3_ composite grating structure on SPR sensing performance. To clearly and effectively reveal the physical correlations between these parameters and the SPR effect, we selected the refractive index range of 1.38 to 1.40 for parametric scanning and performance evaluation. This range lies between the typical aqueous environment and higher-index organic or biochemical solutions. It helps avoid potential interference from water absorption bands in spectral analysis while ensuring stable and significant coupling responses between the surface plasmon polariton mode and the fiber-guided mode, thereby optimally showcasing the subtle impacts of parameter variations. Although the focus is on mechanism exploration, this RI range also holds practical relevance, corresponding to the refractive indices of various biochemical analytes. This establishes a foundation for extending the design principles uncovered here to specific applications such as biomedical diagnostics and environmental monitoring.

## 2. Design and Theoretical Background

### 2.1. Physical Model

The operational foundation of the fiber-optic SPR sensor relies on the efficient excitation of surface plasmon waves (SPWs) at the metal-dielectric interface. This excitation is achieved through the evanescent field component of the guided core mode, which penetrates the fiber cladding when the propagating light undergoes total internal reflection. Subsequently, free electron oscillations are induced on the metal surface when the wavevector of the incident evanescent field matches the propagation constant of the SPWs. This phase-matching condition triggers resonant energy transfer from the core mode to the surface plasmon mode, leading to a characteristic attenuation in the transmitted optical power. Consequently, the output spectrum manifests a distinct absorption peak at the specific resonance wavelength. The fundamental sensing mechanism exploits the linear relationship between the ambient refractive index and the resonance wavelength, enabling precise detection of analyte variations through monitoring the spectral peak shift.

**Figure 1:**
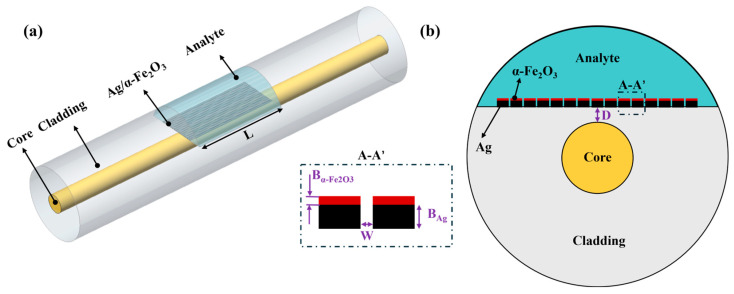
Schematic of SPR sensor based on silver grating-coated hematite (α-Fe_2_O_3_): (**a**) 3D structure; (**b**) Cross-section of SPR sensor.

The sensor configuration, as shown in Figure 1a, employs a side-polished single-mode fiber with a polishing depth of 0.1 μm to create an optimized sensing platform. This D-shaped geometry provides enhanced interaction between the guided light and the external environment through the controlled evanescent field. A sophisticated layered structure is modeled on the polished surface, comprising Ag and α-Fe_2_O_3_ functional layers. The deposition of such layers with precise thickness control, as assumed in our simulation, is achievable using established techniques such as pulsed laser deposition combined with magnetron sputtering [25], which enable the fabrication of thin films with well-defined optical constants and geometries critical for reproducible sensor performance. The fabrication process culminated with the formation of periodic Ag/α-Fe_2_O_3_ grating structures through advanced nanolithography. The critical grating gap width of nanoscale range, while demanding, is achievable using high-resolution nanofabrication methods such as electron-beam lithography (EBL) or focused ion beam (FIB) milling, which are capable of defining features well below this scale [26]. On a curved D-shaped surface, this can be realized through optimized process strategies including precise alignment and resist coating techniques. Although the physical sensor structure is three-dimensional as conceptually illustrated in Figure 1a, the numerical analysis was conducted using a two-dimensional (2D) model. This 2D approach, which models a cross-section of the infinitely long fiber, was selected for its computational efficiency while maintaining accuracy in capturing the essential electromagnetic field distributions and resonance conditions perpendicular to the fiber axis.

The optical fiber parameters were carefully selected to maintain single-mode operation, with core and cladding diameters standardized at 9 μm and 125 μm, respectively. The sensing region length L was optimized to 1 mm [21]. This is because a shorter interaction length diminishes the resonance depth and contrast, impairing reliable detection of the spectral shift, while an excessively long segment introduces heightened propagation losses that can broaden the resonance peak and degrade the signal-to-noise ratio. Figure 1b presents the interface schematic diagram of the SPR sensor. A structure comprising 17 complete grating periods was used to ensure stable electric field distributions. The grating architecture is characterized by several critical parameters: the inter-grating air gap width (W) determines the plasmonic coupling strength, while the respective thicknesses of the α-Fe_2_O_3_ and Ag layers (denoted as B_α-Fe2O3_ and B_Ag_) govern the electromagnetic field distribution and resonance conditions. The residual cladding thickness (D) was precisely controlled to achieve the optimal compromise between resonance sharpness and field enhancement. This comprehensive parameter optimization enables the development of a high-performance SPR sensor with excellent reproducibility and stability.

### 2.2. SPR Phenomenon

The underlying mechanism of SPR relies on a precisely engineered metal-dielectric interface that facilitates the propagation of p-polarized electromagnetic waves, identified as SPWs, along the interfacial boundary. This configuration induces pronounced optical absorption through efficient energy transfer processes occurring during light incidence, manifesting as a measurable reduction in reflected radiation intensity. Consequently, the spectral response demonstrates a distinct resonance feature originating from the evanescent field interaction that exhibits analogous characteristics to SPW. The phase-matching requirement for optimal resonance excitation is mathematically represented by the following fundamental expressions [27]:

(1) $$K_{SPW}=k_{0}{\left(\frac{\varepsilon_{mr}n_{s}^{2}}{\varepsilon_{mr}+n_{s}^{2}}\right)}^{1/2},$$

(2) $$k_{0}=\frac{2\pi }{\lambda },$$

The propagation constant is mathematically represented by the right-hand term in the equation, where ε_mr_ denotes the real component of the metal’s complex dielectric constant (ε_m_). The refractive index (n_s_) of the dielectric sensing layer serves as the fundamental parameter establishing the phase-matching conditions. This methodology demonstrates particular effectiveness for detecting subtle variations in analyte properties due to the exceptional sensitivity of the propagation constant’s matching condition to minute fluctuations in the dielectric environment’s optical characteristics [27].

### 2.3. Fibre Core and Cladding

For the purpose of theoretical modeling and numerical analysis, a step-index single-mode optical fiber configuration was employed, featuring pure fused silica cladding and a germanium-doped silica core (3.1 doped GeO_2_) to achieve the required refractive index contrast. The wavelength-dependent RI(n) characteristics of both fiber core and cladding materials were determined through the Sellmeier dispersion relation, as shown in Equation (3), which provides a physically accurate representation of the material’s optical properties across the investigated spectral range [28].

(3) $$n\left(\lambda \right)={\left(1+\frac{p_{1}\lambda^{2}}{\lambda^{2}-q_{1}^{2}}+\frac{p_{2}\lambda^{2}}{\lambda^{2}-q_{2}^{2}}+\frac{p_{3}\lambda^{2}}{\lambda^{2}-q_{3}^{2}}\right)}^{1/2},$$

where the Sellmeier coefficients are represented by p_1_, p_2_, p_3_, q_1_, q_2_ and q_3_, and the wavelength is represented by λ. The values of these parameters are shown in Table 1 [29].

### 2.4. Dispersion Relationships

Owing to its exceptional plasmonic properties, silver has been extensively employed as the active material in surface plasmon resonance sensors. This noble metal demonstrates a distinct advantage in generating sharper resonance peaks compared to alternative plasmonic materials, thereby providing superior detection accuracy in sensing applications. However, the propensity for silver to undergo oxidation under ambient conditions presents a significant challenge for practical implementation. To mitigate this limitation while simultaneously enhancing sensor performance, a strategic approach involves depositing a protective metal oxide coating on the silver surface, which effectively prevents oxidation while improving the overall sensitivity of the sensing platform [30]. The optical characteristics of the silver layer were mathematically described using the Drude dispersion model [21], as shown in Equations (4) and (5), which accurately represents the wavelength-dependent dielectric function of noble metals. This theoretical framework provides a physical basis for understanding the plasmonic behavior of silver across the investigated spectral range, enabling precise simulation of the interaction between electromagnetic waves and free electrons at the metal-dielectric interface. The model parameters were carefully optimized to reflect the actual optical properties of silver thin films deposited under conditions relevant to sensor fabrication.

(4) $$\varepsilon_{m}\left(\lambda \right)=1-\frac{\lambda^{2}\lambda_{c}}{\lambda_{p}^{2}\left(\lambda_{c}+i\lambda \right)},$$

(5) $$\sqrt{\varepsilon_{m}\left(\lambda \right)}=n_{m}\left(\lambda \right)+jk_{m}\left(\lambda \right),$$

where ε_m_ is the metal’s complex permittivity, k_m_ and n_m_ represent the imaginary and real sections of the index of metal’s refraction, respectively, λ_c_ is the collision wavelength associated with losses, and λ_p_ is the wavelength associated with bulk plasma frequency. In this work, the λ_c_ and λ_p_ of Ag are shown in Table 2 [21].

The refractive index of the α-Fe_2_O_3_ layer was characterized through employing the relationship R = 1 − [T·exp(A)]^1/2^, where A represents the absorption within the layer. The refractive index (n) was subsequently calculated using the approximation *n* = [(1 + R)/(1 − R)] + [((4R)/(1 + R)^2^) − k^2^]^1/2^ [21], where k denotes the extinction coefficient related to the absorption coefficient (α) through the expression k = αλ/4π [31]. This methodological approach enables accurate determination of the optical constants essential for modeling light-matter interactions in the proposed sensor configuration.

The amount of power conveyed via power monitors T assessed by the transmission function was subsequently normalized relative to the incident power of the p-polarization [32].

(6) $$T=\exp\left(-\frac{4\pi }{\lambda_{0}}\cdot imag\left(n_{eff}\right)\cdot L\right),$$

In this work, n_eff_, λ_0_, and L correspond to the effective refractive index of the surface plasmon mode, the wavelength of the incident light, and the length of the sensing region, respectively. The SPR response curve is characterized by plotting the normalized transmitted power as a function of the wavelength. Resonance occurs due to phase matching of the surface plasmon mode with the fundamental mode of the optical fibre, which is manifested as a distinct dip in the transmission spectrum at the resonance condition [21].

### 2.5. Performance Parameters

The performance of the SPR sensor is governed by two fundamental criteria. The primary parameter involves the magnitude of resonance wavelength shift (Δλ_res_) corresponding to a specific variation in the refractive index (Δn) of the sensing layer. The secondary parameter concerns the spectral width of the resonance curve, characterized by the full width at half maximum (F) of the absorption dip. The sensor’s detection capability is primarily determined by the significant resonance wavelength shift that occurs in response to changes in the surrounding dielectric environment. The sensitivity (SI) of an SPR sensor is quantitatively defined as the ratio between the resonance wavelength shift (Δλ_res_) and the refractive index change (Δn), and the detection accuracy (DA) is defined as followed [19]:

(7) $$SI=\frac{\Delta \lambda_{res}}{\Delta n},$$

(8) $$DA=\frac{1}{F},$$

The sensor’s overall performance is quantified through a figure of merit (FOM) that integrates two critical parameters: wavelength sensitivity and spectral resolution characteristics. This comprehensive metric is mathematically defined as the ratio of sensitivity to the full width at half maximum of the resonance curve, as shown in Equation (9), establishing a standardized approach for comparing sensor performance across different configurations [33].

(9) $$FOM=\frac{SI}{F},$$

The sensitivity analysis was performed by varying the external refractive index from 1.38 to 1.40 in increments of Δn = 0.05. For each RI value, the transmission spectrum was simulated. The resonance wavelength for each spectrum was accurately determined by applying a cubic spline interpolation to the simulated data points. The sensitivity was then computed using Equation (7). The full width at half maximum of the resonance dip was directly measured from the interpolated curve for each RI point, and the figure of merit was calculated accordingly using Equation (9).

### 2.6. Numerical Simulation and Mesh Independence Verification

The finite element analysis was conducted using COMSOL Multiphysics 6.3 with the wave optics module. The computational domain was bounded by a perfectly matched layer with a thickness of 600 nm to eliminate non-physical reflections. A mode analysis study was performed on the input fiber port to identify the fundamental mode. Subsequently, in the frequency-domain study, this calculated fundamental mode was selected as the port excitation. The electric field polarization of this mode was oriented perpendicular to the D-shaped polished plane, effectively simulating the incidence of p-polarized light necessary for efficient SPR excitation. During the calculation process, the parameter scanning calculated the optical response within the wavelength range of 500 nm to 1200 nm.

A physics-controlled mesh was utilized, with local refinements applied at the interfaces of the Ag-α-Fe_2_O_3_ grating, where the electromagnetic field gradients are most pronounced. The mesh scheme is shown in Figure S1 and Table S1. Mesh independence was verified by refining the mesh until the shift in sensitivity was negligible. Therefore, the mesh number of 207,922 was chosen for the final simulations to ensure accuracy while managing computational resources.

## 3. Results and Discussions

This work systematically analyzes the impact of each structural parameter on sensor performance. All simulations and analyses are conducted with the variation of the external environmental refractive index within the 1.38 to 1.40 range as the baseline scenario. This range is selected as the analytical framework because it serves as an ideal range for investigating SPR parameter sensitivity: it ensures that the resonance wavelength shift has sufficient dynamic range and measurability while avoiding potential mode mismatch or non-linear effects at extremely high or low RI.

### 3.1. Effects of the Specific Thicknesses of the Ag

**Figure 2:**
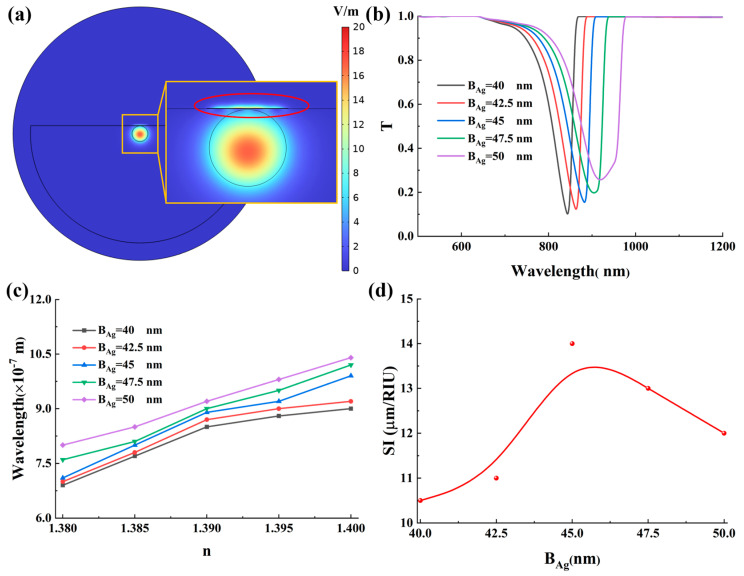
(**a**) The electric field distribution of the Ag/α-Fe_2_O_3_ grating SPR sensor (The SPR phenomenon appeares within the red circle). (**b**) The effect of Ag layer thickness on the transmission spectrum. In this figure, RI = 1.39. (**c**) Relationship between the external RI and resonant wavelength at various Ag layer thickness conditions. (**d**) The effect of Ag layer thickness on the sensitivity.

This section systematically investigates the influence of B_Ag_ on the sensitivity and detection accuracy of the SPR sensor. As B_Ag_ is increased from 40 nm to 50 nm, the corresponding results are summarized in Figure 2. The electric field distribution on the Ag-α-Fe_2_O_3_ grating surface is highly non-uniform, with localized regions of significantly enhanced field intensity, forming on the upper surface, as clearly illustrated in Figure 2a. The periodic grating structure plays a crucial role in defining these hotspots through diffraction. The grating provides the necessary wavevector compensation to bridge the momentum mismatch between the fiber-guided mode and the surface plasmon polaritons (SPPs), thereby selectively enhancing the evanescent field at specific resonant wavelengths. These hot spots arise from the localized surface plasmon resonance effect, which is strongly excited when the wavevector of the incident light matches that of the collective oscillation of free electrons on the metal surface, leading to a resonant energy transfer. Under this phase-matching condition, a substantial portion of the incident light energy is coupled into exciting surface plasmon polaritons, resulting in a pronounced resonance dip in the transmission spectrum, as depicted in Figure 2b. The spectral position and depth of this dip are direct consequences of the grating-mediated, frequency-dependent coupling efficiency. Our analysis reveals that increasing the Ag thickness from 40 nm to 50 nm leads to a gradual increase in the transmission loss at resonance from 0.1 to 0.26, indicating a concomitant weakening of the SPR strength. This attenuation is primarily attributed to increased ohmic losses within the thicker metal layer, which dissipate a larger fraction of the evanescent field energy before it can effectively couple to the surface plasmon polaritons. Concurrently, a redshift of the resonance wavelength is observed, which is associated with the change in the dispersion relation of the surface plasmon polariton mode caused by the increased metal thickness. This shift also implies a modification of the grating’s effective diffraction condition for optimal SPP excitation.

Furthermore, we examined the effect of the external RI variation on this thickness-dependent response, as shown in Figure 2c. Notably, for a fixed B_Ag_, the resonance wavelength exhibits a linear redshift with increasing external RI, a behavior that is fundamentally beneficial for enhancing wavelength-interrogated sensitivity. This linear relationship is governed by the grating-assisted phase-matching condition. Thus, the spectral response explicitly demonstrates how the grating translates RI changes into measurable wavelength shifts via this diffractive coupling mechanism. Comparative analysis indicates that the sensor with B_Ag_ = 45 nm demonstrates the largest resonance wavelength shift over the evaluated RI range, whereas further increasing the thickness beyond this point results in a diminished spectral shift. The sensitivity was evaluated and is presented in Figure 2d. The sensitivity initially increases with B_Ag_, reaching an optimum value for the 45 nm thick Ag-α-Fe_2_O_3_ grating, beyond which it decreases. Although a thicker Ag layer can induce a more substantial redshift, this is often accompanied by a significant broadening of the resonance peak, which consequently degrades the detection accuracy of the sensor.

**Figure 3:**
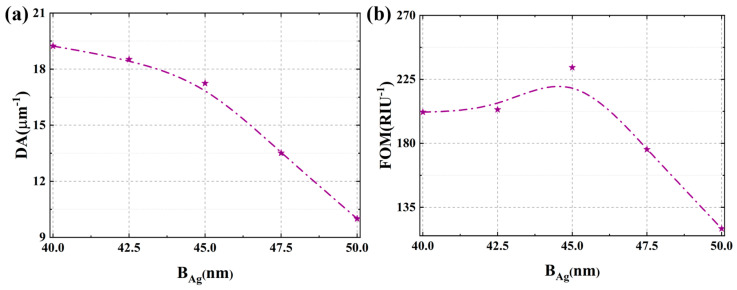
(**a**) The effect of Ag layer thickness on the detection accuracy. (**b**) The effect of Ag layer thickness on the figure of merit.

Figure 3 systematically evaluates the influence of parameter B_Ag_ on the sensor’s detection accuracy and figure of merit. As depicted in Figure 3a, the DA value exhibits a gradual decline with increasing B_Ag_. This deterioration primarily stems from the broadening of the resonance peak, which is induced by enhanced energy dissipation resulting from greater ohmic losses in thicker silver layers. The broader full width at half maximum directly compromises the precision of resonance wavelength determination, thereby reducing DA. In contrast, when assessing the overall performance via the figure of FOM that integrates both sensitivity and resonance linewidth, a distinct trend emerges. As illustrated in Figure 3b, the FOM initially increases with thicker silver layers, reaching an optimum at B_Ag_ = 45 nm, beyond which it declines. This non-monotonic behavior arises from the trade-off between sensitivity enhancement and resonance broadening. While a thicker silver layer strengthens the localized surface plasmon resonance effect and improves SI, excessive thickness leads to significant radiative and ohmic losses, which broaden the resonance peak and degrade the FOM. The optimal performance at B_Ag_ = 45 nm signifies an ideal balance between these competing factors, achieving the highest FOM and underscoring the critical role of precise thickness control in plasmonic sensor design. This optimal thickness of 45 nm corresponds to the condition where the grating structure facilitates the most efficient diffraction-coupled SPP excitation within our spectral window, maximizing the signal-to-noise ratio in the frequency domain. Considering practical fabrication, the sensor exhibits reasonable tolerance around this optimum. A deviation of ±2.5 nm leads to a predictable variation. It can be seen from Table S2 that sensitivity changes by approximately ±1.0 μm/RIU, and FOM varies within about ±15% of its peak value. This smooth, non-catastrophic response confirms that the design is not excessively fragile.

### 3.2. Effects of the Grating Gap Width

**Figure 4:**
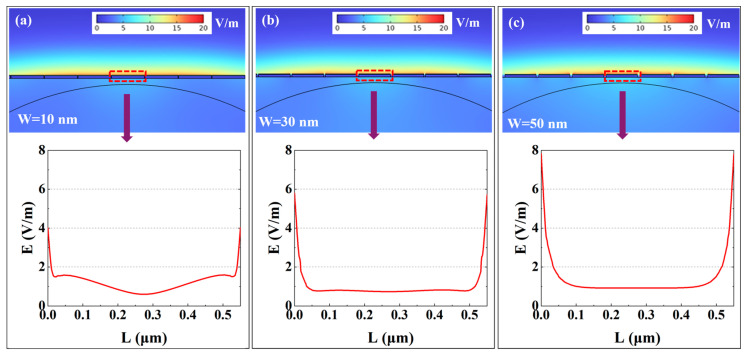
The effect of grating gap on the electric field distribution: (**a**) W = 10 nm; (**b**) W = 30 nm; (**c**) W = 50 nm. The data of the electric field intensity is taken from the surface of the grating within the red box.

This section systematically investigates the influence of W on the sensitivity and detection accuracy of the Ag-α-Fe_2_O_3_-based SPR sensor. The gap width is a critical geometric parameter that directly modulates the diffraction properties of the grating, thereby controlling the wavevector compensation provided to the evanescent field for exciting SPPs. This, in turn, dictates the spectral response of the SPR sensor. Figure 4 illustrates the electric field distribution surrounding the grating structure under varying gap widths. While all configurations with different W values exhibit characteristic electric field enhancement indicative of SPR excitation, their quantitative characteristics demonstrate significant variations, as shown in Figure 4a–c. When the W is minimized to 10 nm, the electric field distribution across the grating surface displays a distinct pattern with intensified regions at the edges and relatively weaker field strength at the center. The maximum field intensity at the grating edges reaches approximately 4 V/m under this configuration. As the gap width increases, a notable enhancement in the peak electric field intensity is observed. Specifically, when W is increased to 50 nm, the electric field amplitude exhibits a substantial enhancement of up to 100% compared to the baseline configuration. The quantitative increase in the maximum field intensity, from 4 V/m at W = 10 nm to 8 V/m at W = 50 nm, serves as a direct metric for the evolving near-field coupling strength. The lower peak field at the minimal gap signifies a regime of strong inter-grating coupling, where energy is distributed in a collective oscillatory mode. The subsequent field enhancement with increasing W quantifies the weakening of this coupling, allowing individual grating elements to resonate more independently and locally concentrate energy more effectively. This phenomenon can be attributed to the transition between different plasmonic coupling regimes. At the minimal gap width of 10 nm, the strong near-field coupling between adjacent silver gratings forms a strongly coupled collective plasmonic oscillation, resulting in the characteristic field distribution pattern observed. The intensified fields at the edges represent localized surface plasmon resonances (LSPRs) enhanced by the sharp geometric features. As the gap width increases, the inter-grating coupling weakens, allowing each grating element to function more independently and support stronger individual LSPR modes. The significant field enhancement at larger gap widths occurs because the reduced parasitic near-field coupling between neighboring gratings enables more efficient light concentration within each individual grating structure. Furthermore, the increased gap dimension permits greater penetration of the incident electromagnetic field, facilitating more effective interaction with the analyte medium and consequently stronger field enhancement. These distinct field distributions for different W are direct spatial manifestations of how the grating’s diffraction efficiency and the resultant SPP excitation vary with its geometry.

**Figure 5:**
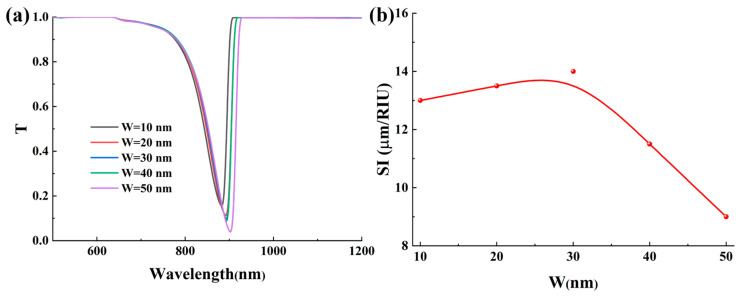
(**a**) The effect of grating gap width on the transmission spectrum. In this figure, RI = 1.39. (**b**) The effect of grating gap width on the sensitivity.

Figure 5a presents the transmission spectra corresponding to different W. As W increases from 10 nm to 50 nm, the transmission loss at the resonance wavelength exhibits a substantial enhancement, with the T value decreasing from 0.15 to 0.04. This significant reduction in transmission indicates a remarkable intensification of the surface plasmon resonance effect, which means that a stronger resonant absorption of light is present. The underlying mechanism for this enhancement can be attributed to the optimized electromagnetic field confinement and improved energy coupling efficiency between adjacent grating elements. At larger W, the individual grating elements can support stronger localized surface plasmon resonances with reduced parasitic near-field coupling and radiation losses, thereby facilitating more efficient energy transfer from the incident light to the plasmonic modes. Thus, the decrease in parasitic inter-grating coupling with increasing W enhances the local energy concentration, which directly manifests as a stronger resonant absorption in the spectrum. Furthermore, a systematic redshift of the resonance wavelength is observed with increasing gap width. This spectral shift provides fundamental advantages for enhancing the sensor’s performance metrics. The redshift phenomenon originates from modifications in the effective refractive index experienced by the surface plasmon polaritons, which is directly influenced by the geometric parameters of the grating structure. As W increases, the reduced coupling between adjacent gratings alters the phase-matching condition, requiring longer wavelengths to satisfy the resonance criterion. This wavelength shift establishes a crucial foundation for substantially improving the sensor’s sensitivity, as it enables a greater spectral displacement per unit change in the ambient refractive index. The combination of enhanced SPR intensity and controlled resonance wavelength tuning through geometric optimization demonstrates a viable pathway for developing high-performance SPR sensors with superior detection capabilities.

Figure 5b illustrates the variation in the SI as a function of W. The calculated SI values initially increase with increasing gap width, reaching a maximum at W = 30 nm, and then decrease for larger W values. This non-monotonic behavior arises from the evolving balance between electromagnetic field confinement and coupling efficiency across different gap widths, indicating a phase-matching relationship that is optimized at specific geometric parameters. Notably, the configuration with W = 30 nm demonstrates the highest capability to induce strong surface plasmon resonance phenomena compared to other gap widths. This optimal performance can be attributed to an improved balance between electromagnetic field confinement and coupling efficiency achieved at this specific geometric parameter. When the gap width is 30 nm, the inter-grating coupling reaches an optimal state where the localized surface plasmon resonances generated at individual grating elements interact constructively without significant radiative losses. This constructive interference enhances the electromagnetic field intensity at the metal-dielectric interface, particularly within the analyte region, thereby strengthening the light-matter interaction.

**Figure 6:**
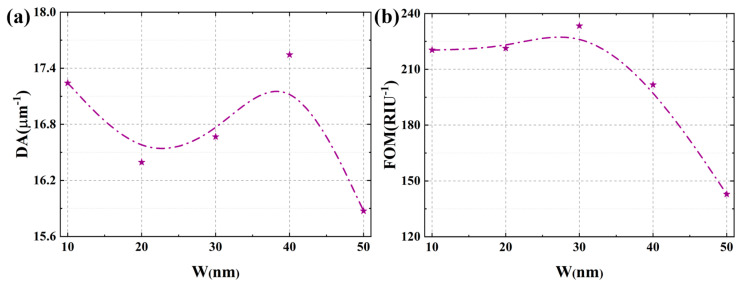
(**a**) The effect of grating gap width on the detection accuracy. (**b**) The effect of grating gap width on the figure of merit.

Figure 6 presents a systematic evaluation of how the W affects the sensor’s DA and FOM. The DA demonstrates a complex non-monotonic response to increasing gap width, characterized by two distinct peaks occurring at W = 10 nm and W = 40 nm, as clearly illustrated in Figure 6a. This behavior primarily stems from the competing effects of integrating coupling strength and radiation losses on the resonance linewidth. When the gap width is minimized to 10 nm, the strong near-field coupling between adjacent gratings forms a strongly coupled collective plasmonic oscillation with confined field distributions, resulting in a relatively narrow resonance peak and consequently higher DA. However, as the gap width increases to approximately 20–30 nm, the weakened inter-grating coupling leads to the formation of individual localized surface plasmon resonances with broader spectral features, thereby reducing the DA. Interestingly, at W = 40 nm, the optimal balance between reduced radiative losses and maintained plasmonic confinement enables a sharper resonance response, explaining the second DA peak. Further increasing the gap width beyond this point diminishes the plasmonic coupling efficiency, resulting in broader resonance peaks and reduced DA. When considering the comprehensive performance metric FOM, which incorporates both the SI and resonance linewidth, a different trend emerges. The FOM exhibits a well-defined optimum at W = 30 nm, as shown in Figure 6b, with performance declining on either side of this maximum. The peak FOM at W = 30 nm signifies the geometric condition where the grating provides an optimal spectral response. It simultaneously achieves high coupling efficiency and a sharp resonance linewidth, which are both frequency-domain attributes governed by the diffraction characteristics of the structure. This optimal configuration achieves an exceptional balance between high electromagnetic field enhancement and minimal radiative losses. The enhanced performance at this specific gap width can be attributed to the optimal plasmonic coupling regime, where the individual grating elements maintain strong localized fields while minimizing parasitic interactions that would otherwise broaden the resonance. Moreover, from a fabrication standpoint, achieving the optimal gap width of W = 30 nm with reasonable precision is critical yet feasible. Our analysis indicates that a deviation of ±5 nm results in a continuous performance shift. It can be seen from Table S2 that the sensitivity varies by approximately ±1 μm/RIU and FOM changes by roughly ±10%. Therefore, targeting a gap width of 30 ± 5 nm constitutes a practical fabrication window that reliably delivers the core performance benefits of the optimized sensor design.

### 3.3. Effects of the Specific Thicknesses of the α-Fe_2_O_3_ Layer

**Figure 7:**
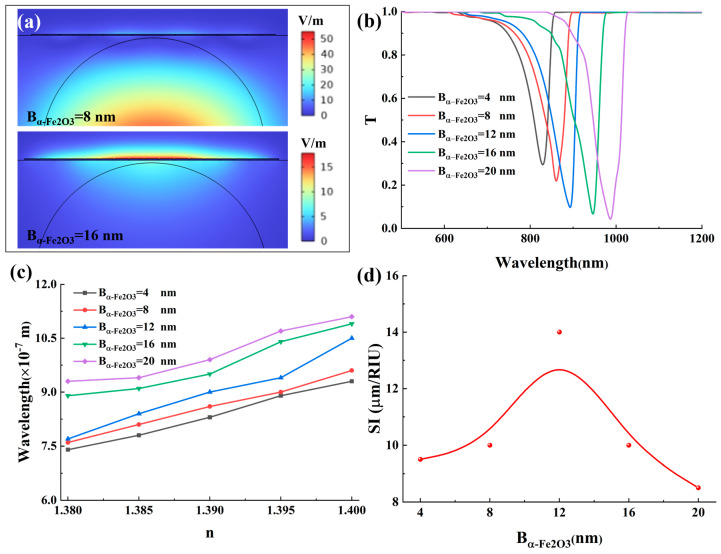
(**a**) The electric field distribution of the Ag/α-Fe_2_O_3_ grating SPR sensor when the hematite layer thickness is different. (**b**) The effect of hematite layer thickness on the transmission spectrum. In this figure, RI = 1.39. (**c**) Relationship between the external RI and resonant wavelength at various hematite layer thickness conditions. (**d**) The effect of hematite layer thickness on the sensitivity.

This section systematically investigates the influence of B_α-Fe2O3_ on the sensing characteristics of the proposed SPR configuration. The α-Fe_2_O_3_ layer, as a dielectric overlay on the Ag grating, critically modifies the local refractive index environment surrounding the periodic metal structure. This alteration directly impacts the grating’s diffraction properties and the resultant wavevector matching condition for SPR excitation, leading to a thickness-dependent spectral response. As illustrated in Figure 7a, progressive thickening of the hematite layer induces significant enhancement of localized electromagnetic fields at the metal-dielectric interface, manifested as intensified hotspot regions with strengthened field confinement. Concurrent spectral analysis in Figure 7b reveals a pronounced redshift of the resonance wavelength with increasing B_α-Fe2O3_, accompanied by measurable broadening of the resonance peak. The observed spectral modifications originate from fundamental modifications in the plasmonic system’s dispersion relations. The redshift phenomenon primarily stems from the dielectric loading effect exerted by the high-refractive-index hematite layer, which effectively increases the effective refractive index experienced by propagating surface plasmon polaritons. This modification necessitates longer wavelengths to satisfy the phase-matching condition for resonance excitation. The simultaneous hotspot enhancement arises from optimized electromagnetic energy confinement within the hematite medium, which reduces radiative losses and strengthens light-matter interactions at the sensing interface. However, this performance enhancement presents a characteristic trade-off. While thicker hematite layers improve electromagnetic confinement and redshift the operational wavelength, they concurrently introduce elevated propagation losses attributable to the material’s intrinsic extinction coefficient. These losses manifest as resonance peak broadening, quantified through increased full width at half maximum values.

This investigation systematically examines the effect of external RI variations on the sensor response relative to B_α-Fe2O3_, as comprehensively illustrated in Figure 7c. A fundamental linear redshift of the resonance wavelength is observed with increasing external RI for any fixed B_α-Fe2O3_ value, demonstrating a characteristic behavior that fundamentally enhances wavelength-interrogated sensitivity. This relationship originates from the proportional increase in the effective refractive index experienced by surface plasmon polaritons at the metal-dielectric interface, which systematically modifies the phase-matching conditions required for resonant excitation. The linear trend for each thickness in Figure 7c is a direct mapping of how the combined effect of the external RI and the fixed hematite thickness dictates the overall phase-matching point via the grating’s diffraction. The slope of these lines, i.e., the sensitivity, is therefore a measure of the spectral responsivity of the grating-coupled SPR system. Comparative analysis reveals that the sensor configuration with B_α-Fe2O3_ = 12 nm exhibits the largest resonance wavelength shift across the evaluated RI range, achieving optimal spectral responsiveness to environmental changes. This maximum performance stems from the ideal electromagnetic field confinement enabled by this specific thickness, which allows optimal overlap between the evanescent field and the analyte medium. Furthermore, a thickness of 12 nm establishes the optimal dielectric environment for the Ag grating to achieve the highest diffraction-coupled energy transfer efficiency within the studied wavelength band, maximizing the spectral shift per RI change. Further increasing the hematite thickness beyond this critical value results in diminished spectral shifts, indicating the onset of suboptimal plasmonic coupling conditions. The sensitivity performance was quantitatively evaluated and presented in Figure 7d, demonstrating a non-monotonic relationship with hematite thickness. The sensitivity initially increases with B_α-Fe2O3_, reaching an optimum value for the 12 nm thick Ag-α-Fe_2_O_3_ grating configuration, beyond which it progressively decreases.

**Figure 8:**
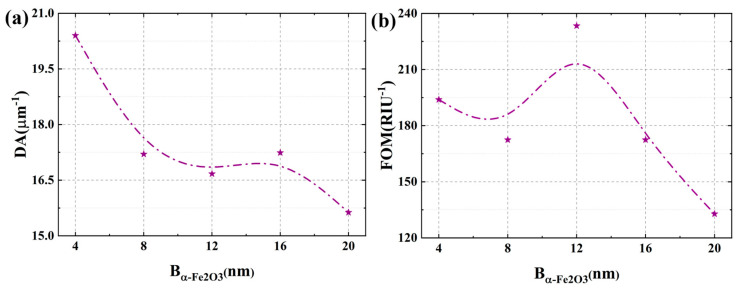
(**a**) The effect of hematite layer thickness on the detection accuracy. (**b**) The effect of hematite layer thickness on the figure of merit.

We systematically evaluate the influence of α-Fe_2_O_3_ layer thickness on the sensor’s DA and FOM. As illustrated in Figure 8a, the DA exhibits a progressive deterioration with increasing B_α-Fe2O3_, primarily attributable to the corresponding broadening of the resonance linewidth. This phenomenon indicates a gradual reduction in detection precision, which stems from increased radiative losses and weakened electromagnetic field confinement at greater hematite thicknesses. The linewidth broadening mechanism is fundamentally linked to the enhanced damping of surface plasmon oscillations caused by the intensified light-matter interactions within the thicker hematite medium. From a spectral perspective, this broadening corresponds to a reduction in the quality factor of the grating-coupled resonance, reflecting a decrease in the sharpness of the frequency-selective response. Comprehensive analysis of the overall sensor performance, as quantified by the FOM metric, reveals a more complex non-monotonic relationship with hematite thickness. The FOM initially decreases as B_α-Fe2O3_ increases to 8 nm, then reaches an optimum value at the critical thickness of 12 nm, as illustrated in Figure 8b, indicating that this specific parameter configuration delivers the best overall performance. The peak in FOM at 12 nm identifies the thickness that yields the best compromise in the frequency domain. It maximizes the spectral shift per RI unit while minimizing the resonance bandwidth, which is the hallmark of an optimally designed diffraction-based sensor. This optimum represents an optimal compromise between resonance sharpness and electromagnetic field enhancement, where the hematite layer provides sufficient dielectric loading to enhance the plasmonic response without introducing excessive optical losses. While thicker hematite layers indeed promote a beneficial redshift of the resonance wavelength due to increased dielectric loading effects, this advantage is counterbalanced by the substantial broadening of the resonance peak. The resulting deterioration in resonance quality ultimately leads to an overall degradation of sensor performance beyond the optimal thickness. This performance trade-off underscores the importance of precise thickness control in optimizing the sensing capabilities of hematite-enhanced SPR configurations. The maximum FOM achieved at B_α-Fe2O3_ = 12 nm demonstrates that this specific thickness provides the ideal balance between sufficient electromagnetic field enhancement and maintained resonance sharpness required for high-performance sensing applications. Moreover, in terms of practical fabrication, the performance exhibits a defined tolerance around this optimum. For instance, a thickness variation of ±2 nm results in a continuous and predictable change: the sensitivity varies between approximately 11.5 and 14.0 μm/RIU, and the FOM fluctuates between about 194 and 233, as shown in Table S2. This corresponds to a performance shift on the order of 15–20% for a ±2 nm error, which is manageable. Therefore, maintaining the α-Fe_2_O_3_ thickness within 12 ± 2 nm is a feasible fabrication target that ensures the core sensing advantages of the design are preserved.

### 3.4. Effects of the Residual Cladding Thickness

**Figure 9:**
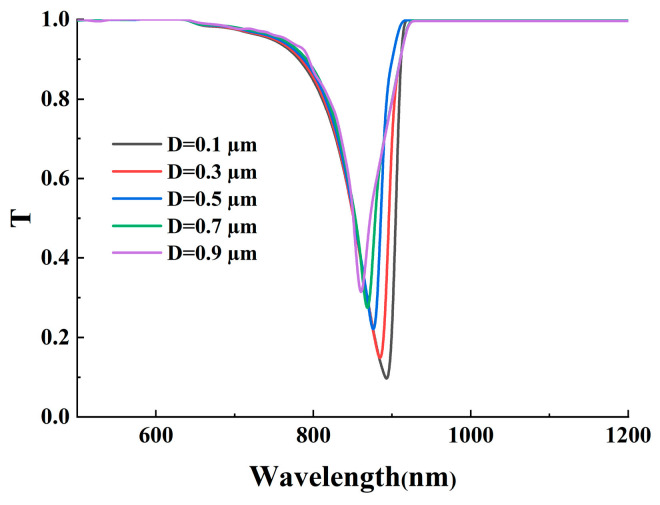
The effect of residual cladding thickness on the transmission spectrum. In this figure, RI = 1.39.

**Figure 10:**
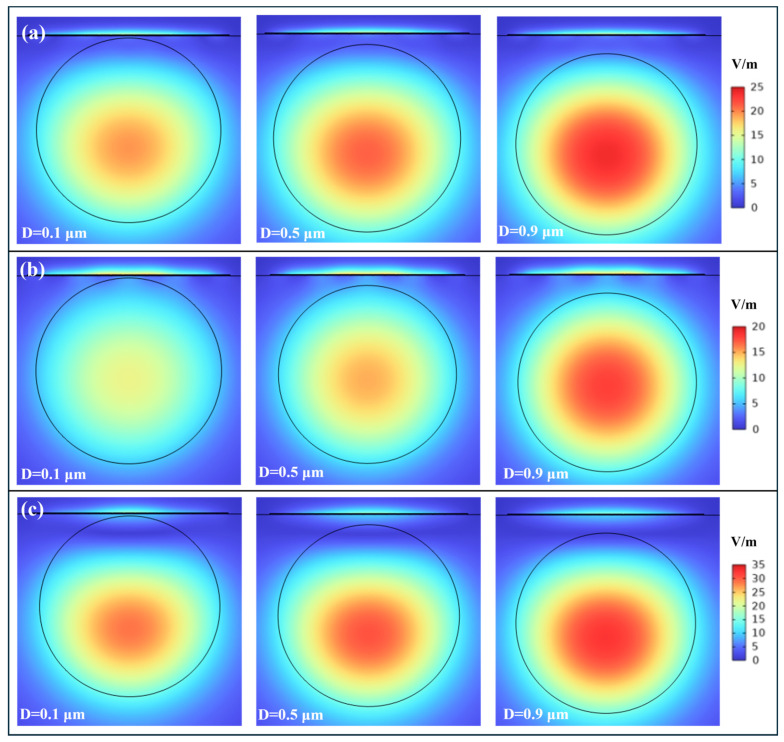
The effect of residual cladding thickness on the electric field distribution: (**a**) RI = 1.38; (**b**) RI = 1.39; (**c**) RI = 1.4.

This section systematically investigates the impact of residual cladding thickness (D) on the operational characteristics of surface plasmon resonance sensors. The residual cladding thickness critically controls the strength and profile of the evanescent field that reaches the Ag-α-Fe_2_O_3_ grating. Since the grating relies on diffraction of this evanescent field to provide the wavevector compensation for SPP excitation, D fundamentally regulates the input to the diffraction process, thereby shaping the overall spectral response. As illustrated in Figure 9, a reduction in D leads to a concurrent deepening and broadening of the SPR curve, indicating an enhancement in the sensor’s sensitivity. The deepening of the resonance dip directly results from enhanced evanescent field coupling between the fiber core and the plasmonic grating as D decreases. This stronger coupling facilitates more efficient energy transfer from the guided core mode to surface plasmon polaritons, increasing the fraction of incident light absorbed at the metal-dielectric interface and thus deepening the resonance. Enhanced energy transfer is a consequence of improved diffraction efficiency of the grating when driven by a stronger evanescent field, leading to a more pronounced excitation of SPPs at the resonance wavelength. This morphological change in the resonance curve is accompanied by a broadening of the FWHM. The broadening mechanism is attributed to increased radiative losses and potential inter-modal interference under conditions of very strong evanescent coupling when D is small. The observed weakening in SPR intensity with thicker residual cladding primarily stems from the exponential decay characteristic of evanescent fields in optical waveguides. As the residual cladding thickness increases, the penetration depth of the evanescent field becomes insufficient to effectively reach and excite surface plasmons at the metal-dielectric interface. Consequently, the grating operates with a weaker driving field, reducing the effective strength of the diffractive coupling and thus the overall SPR excitation efficiency, which manifests as a shallower resonance dip. The metallic layer requires a critical energy density to sustain strong plasmonic oscillations, which cannot be maintained when the evanescent field intensity drops below a certain threshold due to excessive cladding thickness. This physical mechanism finds further confirmation in Figure 10, where increased residual cladding thickness correlates with enhanced energy confinement within the fiber core and consequently reduced SPR excitation at the grating interface. The data consistently demonstrates that thicker residual cladding leads to stronger energy localization in the central fiber region while generating weaker surface plasmon resonance, a trend that remains consistent across different external refractive index conditions. Furthermore, variations in D also influence the resonance wavelength shift, a key determinant of sensitivity. A decrease in D alters the phase-matching condition by modifying the effective refractive index of the core-guided mode and the interaction strength of the evanescent field with the plasmonic layer. This typically induces a redshift in the resonance wavelength, enhancing the spectral displacement per unit change in the surrounding refractive index. The compromised evanescent field strength ultimately limits the sensor’s ability to detect subtle refractive index variations, particularly affecting the sensitivity performance in biological and chemical sensing applications where precise measurements are crucial. Furthermore, the phenomenon of SPR intensity attenuation with increasing D demonstrates remarkable consistency across various refractive index conditions, as shown in Figure 10a–c.

**Figure 11:**
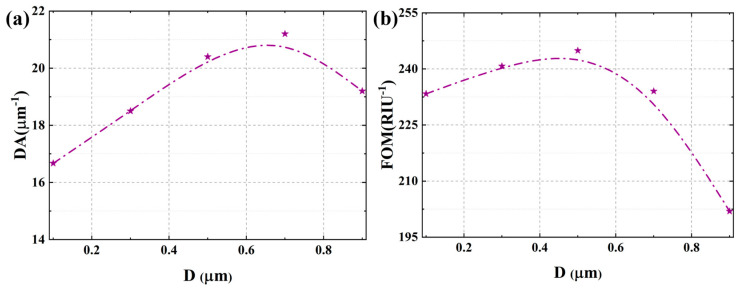
(**a**) The effect of residual cladding thickness on the detection accuracy. (**b**) The effect of residual cladding thickness on the figure of merit.

Systematic evaluation of D reveals its profound impact on both DA and FOM of the SPR sensor. As clearly demonstrated in the analytical results, the DA exhibits a distinct non-monotonic relationship with increasing cladding thickness, initially enhancing before subsequent degradation, with optimal detection precision achieved at D = 0.7 μm, as shown in Figure 11a and Table S3. This behavior originates from the competing effects of D on resonance linewidth. At very small D, strong coupling leads to significant broadening, degrading DA. As D increases to an optimal value (~0.7 μm), the coupling is moderated, reducing radiative losses and yielding a sharper resonance, thereby maximizing DA. Beyond this point, further increase in D weakens coupling excessively, leading again to a degradation of the resonance signal quality. The optimal DA at D = 0.7 μm therefore represents the cladding thickness that minimizes losses and optimizes the spectral purity of the grating-diffracted resonance, yielding the narrowest linewidth. In contrast, a comprehensive evaluation of the overall sensor performance was conducted using FOM, which systematically incorporates both sensitivity characteristics and resonance linewidth measurements. This integrated analytical approach reveals a distinct optimal operational point occurring at a residual cladding thickness of D = 0.5 μm, as shown in Figure 11b and Table S3, demonstrating a significant shift from the individual parameter optima observed in previous analyses. This divergence arises from the fundamental trade-off between signal sharpness and responsiveness to refractive index variations. The underlying physical reason for the separation of optima lies in the different definitions and physical emphases of DA and FOM. DA is defined as the inverse of F, focusing solely on the resonance peak’s spectral confinement. Therefore, D = 0.7 μm represents the condition where evanescent coupling is tuned to minimize radiative and dissipative losses, producing the narrowest possible resonance and thus the highest DA. On the other hand, FOM is defined as SI divided by F. It therefore demands a balance between a large resonance wavelength shift and a narrow resonance linewidth. While D = 0.7 μm reduces F, the relatively weaker coupling at this thickness results in a sub-optimal wavelength sensitivity. Conversely, at D = 0.5 μm, the evanescent coupling is stronger. This significantly enhances the interaction between the guided light and the analyte, leading to a greater perturbation of the phase-matching condition and consequently a larger resonance wavelength shift per unit RI change. This stronger coupling at D = 0.5 μm provides a more robust evanescent field to drive the grating’s diffraction, enhancing the efficiency with which RI changes are converted into spectral shifts (higher SI), which is the numerator in the FOM. Although this stronger coupling slightly increases the F compared to the D = 0.7 μm case, the net gain in sensitivity is proportionally greater. While the thicker cladding (0.7 μm) provides superior resonance definition, the moderately thinner configuration (0.5 μm) maintains stronger plasmonic coupling efficiency, thereby enhancing the wavelength shift per unit refractive index change.

### 3.5. Performance Comparison

The performance of SPR sensor is benchmarked against contemporary SPR designs, as shown in Table 3. The foundational work [19], which shares the Ag–α-Fe_2_O_3_ material system, reported a sensitivity of 6.4 µm/RIU. Our systematic geometric optimization, particularly of the grating gap, has more than doubled this value to 14 µm/RIU, confirming the significant performance headroom attainable through refined design even within an established material platform. When compared to other recent configurations employing diverse materials, the merit of our optimized design becomes clear. While hybrid structures incorporating materials like Ti_3_C_2_T_x_-MXene [20] or complex Au/Ag/MoS_2_ stacks [21] can achieve high sensitivities, they often involve more elaborate fabrication or less stable material combinations. Our sensor demonstrates a sensitivity superior to several such designs while retaining the practical advantages of a simpler, potentially more robust material system centered on Ag and α-Fe_2_O_3_. In summary, substantial performance gains for SPR sensors can be achieved not only through the introduction of novel materials but also through a rigorous, system-level re-optimization of geometric parameters in existing, promising platforms.

## 4. Conclusions

This study presents a systematic numerical investigation that elucidates the intricate design principles and performance trade-offs governing a D-shaped optical fiber SPR sensor with an Ag-α-Fe_2_O_3_ composite grating. Beyond merely identifying optimal parameters, the analysis reveals that the ultimate sensor performance stems from a delicate synergy and balance among multiple geometric dimensions. The core findings are synthesized below to provide a clear and actionable framework for the design of high-performance, grating-coupled fiber SPR sensors.

The foundation of the optimized design is established by the Ag layer, whose thickness is a pivotal parameter governing fundamental plasmonic activity. An optimal thickness of 45 nm was determined to strike a critical balance between achieving a strong localized surface plasmon resonance effect, which induces a substantial resonant redshift for high sensitivity, and minimizing the detrimental ohmic and radiative losses that cause resonance broadening and degrade detection accuracy, thereby yielding the highest figure of merit.

Building upon this plasmonic foundation, the grating gap width critically modulates the near-field coupling regime between adjacent elements. An optimal width of 30 nm was found to facilitate a state where individual grating elements support strong, independent LSPR modes while effectively suppressing parasitic inter-element coupling and radiative losses prevalent at smaller gaps. This configuration enables the most efficient constructive interference of localized fields at the sensing interface, leading to pronounced field enhancement and maximal sensitivity.

Complementing the metallic grating, the α-Fe_2_O_3_ overlay serves the dual function of a protective capping layer and a high-refractive-index dielectric modulator. Its optimal thickness of 12 nm provides sufficient dielectric loading to effectively redshift the resonance and enhance field confinement at the interface, thereby boosting sensitivity, without introducing excessive propagation losses from the material’s intrinsic extinction coefficient that would otherwise broaden the resonance and rapidly degrade performance.

Finally, the system’s coupling efficiency is governed by the residual cladding thickness, which controls the evanescent field strength incident on the grating. A fundamental trade-off exists between resonance sharpness and coupling strength. While a thickness of 0.7 µm optimizes phase-matching for the sharpest resonance dip, the overall system performance is maximized at a thinner cladding of 0.5 µm. At this thickness, stronger evanescent coupling prioritizes a larger resonance wavelength shift per unit refractive index change, accepting a marginally broader resonance linewidth as a trade-off.

Beyond identifying the optimal values, the analysis reveals their relative sensitivity to fabrication tolerances, which is crucial for experimental realization. The α-Fe_2_O_3_ layer thickness and the grating gap width emerge as the most critical parameters, requiring stringent control due to their sharp influence on the balance between dielectric loading/coupling and optical loss. In contrast, the Ag layer thickness and the residual cladding exhibit more moderate tolerances. It should be noted that the simulated high performance is based on ideal conditions. In practical fabrication, factors such as deviations in material optical constants, interfacial roughness, and geometric imperfections may lead to resonance broadening and a redshift in the resonance wavelength compared to theoretical predictions. The future work will focus on achieving high-precision nano-manufacturing, approaching the results of simulation design, and combining SPR technology with acoustic pre-treatment [34,35] to carry out biochemical detection applications.

## Figures and Tables

**Table 1:** Sellmeier coefficients for the fibre core and cladding.

Coefficients	p_1_	p_2_	p_3_	q_1_ (μm)	q_2_ (μm)	q_3_ (μm)
Cladding	0.6961663	0.4079426	0.8974794	0.0684043	0.1162414	9.896161
Core	0.7028554	0.4146307	0.8974540	0.0727723	0.1143085	9.896161

**Table 2:** Dispersion coefficients for Ag.

Dispersion Coefficients (μm)	Ag
λ_c_	0.14541
λ_p_	17.614

**Table 3:** Performance comparison.

Material	RI Range	Sensitivity (μm/RIU)	Ref.
Au + 7% GeO_2_ doped SiO_2_	1.0–1.5	2	[19]
Au/Ti_3_C_2_T_x_-MXene	1.390–1.415	64.6	[20]
Ag-α-Fe_2_O_3_	1.33–1.39	6.4	[21]
Ag + Au + MoS_2_	1.33–1.40	11.78	[23]
Au	1.47–1.52	14.78	[24]
Ag-α-Fe_2_O_3_	1.38–1.40	14	This work

## Data Availability

The original contributions presented in this study are included in the article/Supplementary Materials. Further inquiries can be directed to the corresponding authors.

## Supplementary Materials

The following supporting information can be downloaded at https://www.mdpi.com/article/10.3390/mi17020183/s1, Figure S1: Meshing of the computational domain; Table S1: Mesh scheme of the computational domain; Table S2: The influence of manufacturing errors of B_Ag_, B_α-Fe2O3_ and W on the performance of SPR sensors; Table S3: SI, F, DA and FOM under different D conditions.

## References

[1] Li N., Cheng C., Wu D., Song Z., Wang B., Li G., Yang F. (2025). Immunofluorescent Analysis of Exosomes Using a Microchip Filled with Transparent Antibody-Conjugated Beads for Breast Cancer Liquid Biopsy. Anal. Chim. Acta.

[2] Zhou M., Li J., Yuan S., Yang X., Lu J., Jiang B. (2024). A Centrifugal Microfluidic System for Automated Detection of Multiple Heavy Metal Ions by Aptamer-Based Colorimetric Assay. Sens. Actuators B Chem..

[3] Peng T., Qiang J., Yuan S. (2023). Investigation on a Cascaded Inertial and Acoustic Microfluidic Device for Sheathless and Label-Free Separation of Circulating Tumor Cells. Phys. Fluids.

[4] Yuan S. (2024). Indirect Detection of Lead(II), Cadmium(II) and Mercury(II) on a Microfluidic Electrophoresis Chip. Anal. Methods.

[5] Chen W., Cao R., Su W., Zhang X., Xu Y., Wang P. (2021). Lab on a Chip with a Chitosan Modified Shuttle Flow Microchip. Lab Chip.

[6] Yuan S., Zhang P., Yang S., Chen J., Mu X., Wu C., Deng J. (2025). Targeted Cellular Depletion in an Immune-Liver-on-a-Chip Platform Elucidates Cell-Type-Specific Heterogeneity in Drug-Induced Hepatotoxicity. Commun. Biol..

[7] Zou B., Lv K., Xu J., Feng S., Zhang G. (2025). High-Sensitivity SERS Based on One-Dimensional TiO_2_/Ag Nanowires. Phys. B Condens. Matter.

[8] Substrates G.A.Z., Bandarenka H., Burko A., Laputsko D., Dronina L., Kovalchuk N., Podelinska A., Shapel U., Popov A.I., Bocharov D. (2023). Ultraviolet Exposure Improves SERS Activity of Graphene-Coated Ag/ZrO_2_ Substrates. Crystals.

[9] Das S., Devireddy R., Gartia M.R. (2023). Surface Plasmon Resonance (SPR) Sensor for Cancer Biomarker Detection. Biosensors.

[10] Mahmoudpour M., Ezzati Nazhad Dolatabadi J., Torbati M., Homayouni-Rad A. (2019). Nanomaterials Based Surface Plasmon Resonance Signal Enhancement for Detection of Environmental Pollutions. Biosens. Bioelectron..

[11] Beeram R., Vepa K.R., Soma V.R. (2023). Recent Trends in SERS-Based Plasmonic Sensors for Disease. Biosensors.

[12] Masson J.F. (2017). Surface Plasmon Resonance Clinical Biosensors for Medical Diagnostics. ACS Sens..

[13] Yesudasu V., Pradhan H.S., Pandya R.J. (2021). Recent Progress in Surface Plasmon Resonance Based Sensors: A Comprehensive Review. Heliyon.

[14] Aljbar N.A., Mahdi B.R., Khalid A.H., Attallah A.H., Abdulwahid F.S., Haider A.J. (2025). Enhanced Surface Plasmon Resonance (SPR) Fiber Optic Sensor for Environmental Monitoring: A Coreless Fiber–Based Design. Plasmonics.

[15] Chauhan M., Kumar Singh V. (2021). Review on Recent Experimental SPR/LSPR Based Fiber Optic Analyte Sensors. Opt. Fiber Technol..

[16] Divya J., Selvendran S. (2025). Performance Evaluation of D-Shaped Photonic Crystal Fiber Based SPR Sensors with Different Plasmonic Materials: A Comparative Analysis. Results Eng..

[17] Liu W., Hu C., Zhou L., Yi Z., Liu C., Lv J., Yang L., Chu P.K. (2022). A Square-Lattice D-Shaped Photonic Crystal Fiber Sensor Based on SPR to Detect Analytes with Large Refractive Indexes. Phys. E Low-Dimens. Syst. Nanostructures.

[18] Uwais M., Rastogi V. (2025). Cu—Grating and Graphene—Assisted SPR—Based Refractive Index Sensor. Plasmonics.

[19] Osamah S., Fakhri M.A., Alwahib A.A., Salim E.T., Ibrahim R.K., Mohammed A.F.A., Gopinath S.C.B., Qaeed M.A., Ibrahim H.I., Ahmed A.S. (2024). A Novel Design of Symmetrical Grating Built on D-Shaped Optical Fi Ber Sensor-Based Surface Plasmon Resonance. Adv. Nat. Sci. Nanosci. Nanotechnol..

[20] Mu R., Wan H., Shi W., Liang H., Lou Y. (2023). Design and Theoretical Analysis of High-Sensitive Surface Plasmon Resonance Sensor Based on Au/Ti 3C2Tx-MXene Hybrid Layered D-Shaped Photonic Crystal Fiber. IEEE Sens. J..

[21] Kadhim R.A., Yuan L., Xu H., Wu J., Wang Z. (2020). Highly Sensitive D-Shaped Optical Fiber Surface Plasmon Resonance Refractive Index Sensor Based on Ag-α-Fe_2_O_3_Grating. IEEE Sens. J..

[22] Suchikova Y., Nazarovets S., Konuhova M., Popov A.I. (2025). Binary Oxide Ceramics (TiO_2_, ZnO, Al_2_O_3_, SiO_2_, CeO_2_, Fe_2_O_3_, and WO_3_) for Solar Cell Applications: A Comparative and Bibliometric Analysis. Ceramics.

[23] Dogan Y., Erdogan I. (2023). Highly Sensitive MoS_2_/Graphene Based D-Shaped Optical Fiber SPR Refractive Index Sensor with Ag/Au Grated Structure. Opt. Quantum Electron..

[24] Yan X., Fu R., Cheng T., Li S. (2021). A Highly Sensitive Refractive Index Sensor Based on a V-Shaped Photonic Crystal Fiber with a High Refractive Index Range. Sensors.

[25] Merhan Muğlu G., Şenay V., Saritaş S., Abdolahpour Salari M., Kundakçi M. (2025). The Structural, Optical, Topographical, and H_2_ Sensing Characteristics of a Zn-Doped Fe_2_O_3_ Thin Layer Deposited via DC & RF Magnetron Co-Sputtering Method. J. Mater. Sci. Mater. Electron..

[26] Jiang X., Gu Q., Wang F., Lv J., Ma Z., Si G. (2013). Fabrication of Coaxial Plasmonic Crystals by Focused Ion Beam Milling and Electron-Beam Lithography. Mater. Lett..

[27] Sharma A.K., Jha R., Gupta B.D. (2007). Fiber-Optic Sensors Based on Surface Plasmon Resonance: A Comprehensive Review. IEEE Sens. J..

[28] Akouibaa A., Akouibaa A., Masrour R., Benhamou M., Rezzouk A., Heryanto H. (2024). Numerical Simulation of Early Detection of Cancer Cells Using a D-Shaped Fiber-Optic Biosensor Based on Surface Plasmon Resonance. J. Clust. Sci..

[29] Nayak J.K., Jha R. (2017). Numerical Simulation on the Performance Analysis of a Graphene-Coated Optical Fiber Plasmonic Sensor at Anti-Crossing. Appl. Opt..

[30] Al-Qazwini Y., Noor A.S.M., Arasu P.T., Sadrolhosseini A.R. (2013). Investigation of the Performance of an SPR-Based Optical Fiber Sensor Using Finite-Difference Time Domain. Curr. Appl. Phys..

[31] Kumar P., Rawat N., Hang D.R., Lee H.N., Kumar R. (2015). Controlling Band Gap and Refractive Index in Dopant-Free α-Fe_2_O_3_ Films. Electron. Mater. Lett..

[32] Banerjee A., Thangaraj J. (2025). SPR-Based Refractive Index Sensor Design with Grated Au-ZnS for Dengue Detection Using Machine Learning. Spectrochim. Acta—Part A Mol. Biomol. Spectrosc..

[33] Villarim M.R., Belfort D.R., de Souza C.P. (2023). A Surface Plasmon Resonance (SPR)-Based Biosensor Simulation Platform for Performance Evaluation of Different Constructional Configurations. Coatings.

[34] Peng T., Zhou M., Yuan S., Fan C., Jiang B. (2022). Numerical Investigation of Particle Deflection in Tilted-Angle Standing Surface Acoustic Wave Microfluidic Devices. Appl. Math. Model..

[35] Peng T., Lin X., Yuan S., Zhou M., Jiang B., Jia Y. (2023). Mixing Enhancement in a Straight Microchannel with Ultrasonically Activated Attached Bubbles. Int. J. Heat Mass Transf..

