# Supplementary material for: Enhanced Sensitivity in D-Shaped Optical Fiber SPR Sensor via Ag-α-Fe2O3 Grating
Source: Micromachines (Basel). 2026 Jan 29;17(2):183. doi: 10.3390/mi17020183 (PMC12943643; doi:10.3390/mi17020183)
Supplement: Supplementary file 1 [file micromachines-17-00183-s001.zip › micromachines-4064978-supplementary.pdf]

Enhanced sensitivity in D-Shaped optical fiber SPR sensor via Ag-α-Fe_2O_3 grating

Shuai Yuan [1, 2,*], Bingyang Yuan [1,3], Jiu Deng [1, *]

1 School of Health and Life Sciences, University of Health and Rehabilitation Sciences, Qingdao, Shandong, 266113, China

2 Ocean Decade International Cooperation Center (ODCC), Qingdao, Shandong, 266520, China

3 College of Shipbuilding Engineering, Harbin Engineering University, Harbin, Heilongjiang, 150001, China

**Correspondence:* yuanshuai1006@hotmail.com; dengjiu@uhrs.edu.cn

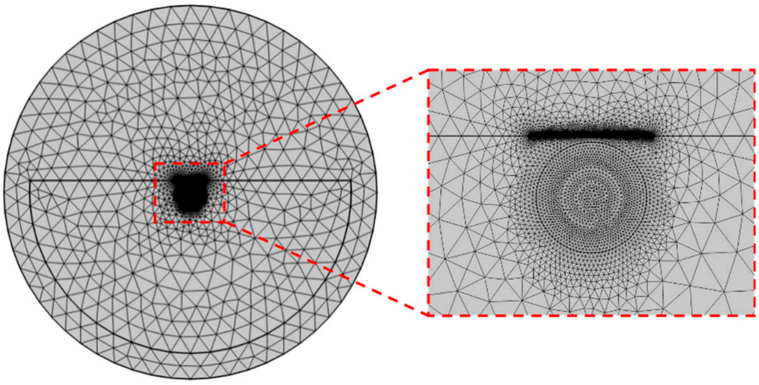

Figure. S1 Meshing of the computational domain.

Table. S1 Mesh scheme of the computational domain.

Mesh scheme	Mesh number	SI	$\frac{\lvert \Delta SI_{k+1} - \Delta SI_k \rvert}{\Delta SI_k} \times 100\%$
1	19,330	19.2	-
2	145,436	15.2	20.8%
3	207,922	14.0	7.9%
4	445,364	13.6	2.9%

Table. S2 The influence of manufacturing errors of B_{Ag}, $B_{\alpha\text{-}Fe_2O_3}$ and W on the performance of SPR sensors.

Parameters	Range (nm)	SI	FOM
B_{Ag}	42.5	11.0	203
	45	14.0	233
	47.5	13.0	175
$B_{\alpha\text{-}Fe_2O_3}$	10	11.5	194
	12	14.0	233
	14	11.8	188
W	25	13.8	215
	30	14.0	233
	35	12.5	210

Table. S3 SI, F, DA and FOM under different D conditions.

D (μm)	△λ (μm)	SI	F (μm)	DA	FOM
0.1	0.070	14.0	0.060	16.67	233.33
0.3	0.065	13.1	0.054	18.51	240.74
0.5	0.060	11.9	0.049	20.41	244.89
0.7	0.055	11.0	0.047	21.28	234.04
0.9	0.053	10.5	0.052	19.23	201.92
